# Supplementary material for: Defocus and magnification dependent variation of TEM image astigmatism
Source: Sci Rep. 2018 Jan 10;8:344. doi: 10.1038/s41598-017-18820-x (PMC5762780; doi:10.1038/s41598-017-18820-x)
Supplement: Supplementary file 1 — Supplementary Information [file 41598_2017_18820_MOESM1_ESM.pdf]

Defocus and magnification dependent variation of TEM image astigmatism

Rui Yan, Kunpeng Li, Wen Jiang

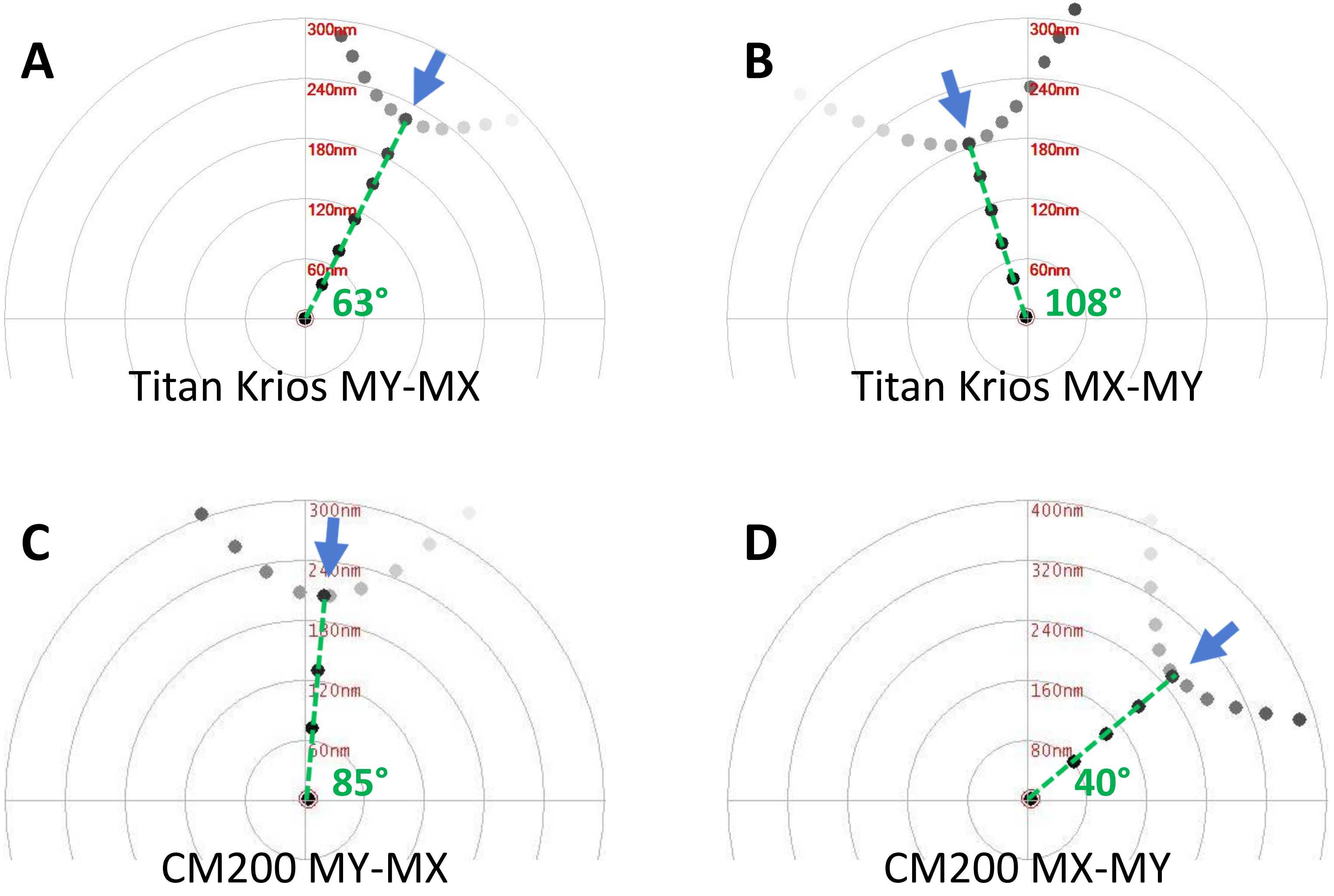

Fig. S1 Performance of *s²stigmator* method and the single-pass tuning strategy. (A, B) Two screenshots of the trajectory from Titan Krios microscope obtained at 1000 nm defocus, a nominal magnification of 22,500X and 25 e/Å^2 dose with images recorded on a Gatan K2 Summit direct electron detector operated at counted mode using 15 e/pixel/second dose rate and 3s exposure time. Stigmator MY was adjusted first (arc-like segment) and then MX was adjusted (straight segment) in (A) while the opposite order (i.e. MX first and then MY) was used in (B). (C, D) Two screenshots of trajectory from CM200 microscope obtained at 1700 nm defocus, a nominal magnification of 115,000X and 40 e/Å^2 dose with images recorded on a Gatan UltraScan 4k CCD with 3s exposure time. Stigmator MY was adjusted first (arc-like segment) and then MX was adjusted (straight segment) in (C) while the opposite order (i.e. MX first and then MY) was used in (D). The wide blue arrow indicates the optimal point with minimal astigmatism in the arc-like segment of each trajectory. The green dash line in each plot represents the angle of the straight trace segment in the trajectory, and the exact angle is marked in green next to the green dash line. Both sequences of stigmator adjustment (MY first, then MX or MX first, then MY) on the same instrument are able to minimize astigmatism with the similar shape of trajectories with 45° angular offset (63° in A v.s. 108° in B, and 85° in C v.s. 40° in D).

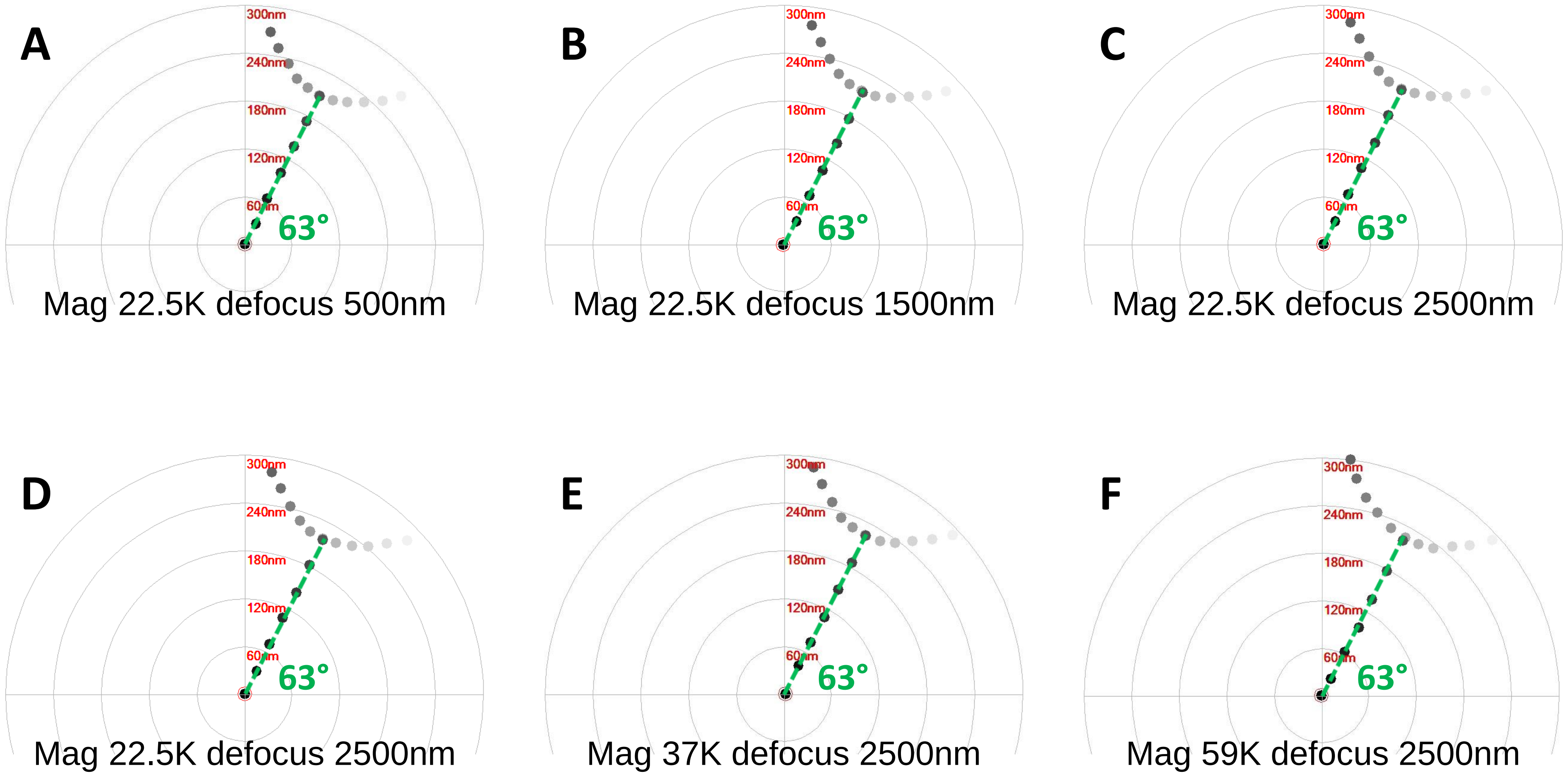

Fig. S2 Representative trajectories of astigmatism correction at varying defocuses and magnifications on Titan Krios microscope. (A-C) The screenshots of the trajectories acquired at a nominal magnification of 22,500X and defocus 500 nm (A), 1500 nm (B) and 2500 nm (C), respectively. (D-F) The screenshots of the trajectories acquired at a nominal magnification of 22,500X (D), 37,000X (E) and 59,000X (F), respectively, and defocus 2500 nm. The green dash line in each plot represents the angle of the straight trace segment in the trajectory, and the exact angle is marked in green next to the green dash line. In these six screenshots of trajectories obtained from Titan Krios when correcting astigmatism at different defocuses (A-C) and different magnifications (D-F), the trajectories are very similar and all consistently led to correction of astigmatism at a wide range of defocuses and magnifications. The angle of the straight trace segment (63°) corresponds to the 2nd stigmator used in this single-pass strategy, i.e. stigmator MX here. This angle is determined by the angular position of the stigmators, e.g. octupole objective lens stigmator [10,11].

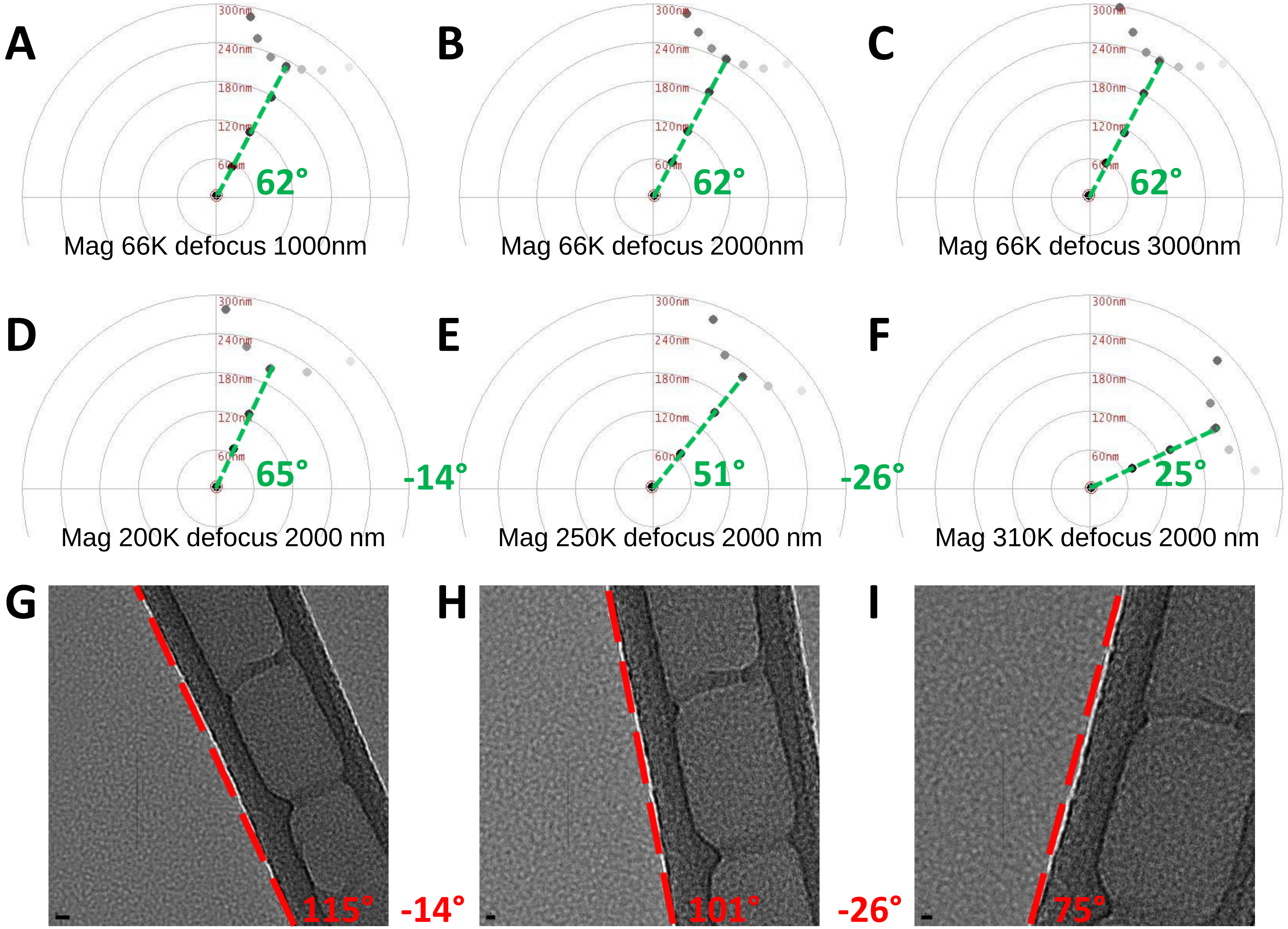

Fig. S3 Representative trajectories of astigmatism correction at varying defocuses and magnifications on CM200 microscope. (A-C) The screenshots of the trajectories acquired at a nominal magnification of 66,000X and defocus 1000 nm (A), 2000 nm (B) and 3000 nm (C), respectively. The green dash line in each plot represents the angle of the straight trace segment in the trajectory, and the exact angle is marked in green next to the green dash line. The angles of the straight trace segments are the same (62° in A-C). (D-F) The screenshots of the trajectories acquired at a nominal magnification of 200,000X (D), 250,000X (E) and 310,000X (F), respectively, and defocus 2000 nm. The angles of the straight trace segments are 65°, 51° and 25°, respectively. And the difference between two adjacent trajectories are 14° and 26°. (G-I) The real space images collected at a nominal magnification of 200,000X (G), 250,000X (H) and 310,000X (I), respectively. The rotation of the red dash line represents the rotation of the image in real space with the change of magnifications. The angles of the red dash lines are 115°, 101° and 75°, respectively. And the angular difference between two adjacent images are 14° and 26°, in agreement with those of the trajectories shown in (D-F). Similar to Fig. S2, the use of different defocuses for astigmatism correction on CM200 does not have a significant influence on the shape of the trajectories (A-C). However, the switch of magnification does have an effect on the orientation of the trajectories (D-F). It is noted that the trajectories turn clockwise when magnification increases (D-F), which is consistent with the rotation of real images at the same set of magnifications (G-I). Thus, we attribute the rotation of trajectories at different magnifications to the imperfect implementation of the rotation-free imaging function on CM200.
